# Novel fusion of GLP-1 with a domain antibody to serum albumin prolongs protection against myocardial ischemia/reperfusion injury in the rat

**DOI:** 10.1186/1475-2840-12-148

**Published:** 2013-10-14

**Authors:** Weike Bao, Lucy J Holt, Rob D Prince, Gavin X Jones, Karpagam Aravindhan, Mathew Szapacs, April M Barbour, Larry J Jolivette, John J Lepore, Robert N Willette, Elena DeAngelis, Beat M Jucker

**Affiliations:** 1 Heart Failure Discovery Performance Unit, Metabolic Pathways and Cardiovascular Therapy Area Unit, GlaxoSmithKline, King of Prussia, PA 19406, USA; 2 Innovation BDU, Biopharm Research, Cambridge, UK; 3 DMPK, Platform Technology and Science, King of Prussia, PA 19406, USA; 4 Clinical Pharmacology Modelling and Stimulation, King of Prussia, PA 19406, USA; 5 Preclinical and Translational Imaging, Platform Technology and Science, GlaxoSmithKline, King of Prussia, PA 19406, USA

**Keywords:** Glucagon-like peptide-1 (GLP-1), Exendin-4, Anti-albumin-binding domain antibody, Rat, Cardioprotection

## Abstract

**Background:**

Glucagon-like peptide-1 (GLP-1) and its mimetics reduce infarct size in the setting of acute myocardial ischemia/reperfusion (I/R) injury. However, the short serum half-life of GLP-1 and its mimetics may limit their therapeutic use in acute myocardial ischemia. Domain antibodies to serum albumin (AlbudAbs) have been developed to extend the serum half-life of short lived therapeutic proteins, peptides and small molecules. In this study, we compared the effect of a long acting GLP-1 agonist, DPP-IV resistant GLP-1 (7–36, A8G) fused to an AlbudAb (GAlbudAb), with the effect of the GLP-1 mimetic, exendin-4 (short half-life GLP-1 agonist) on infarct size following acute myocardial I/R injury.

**Methods:**

Male Sprague–Dawley rats (8-week-old) were treated with vehicle, GAlbudAb or exendin-4. Myocardial ischemia was induced 2 h following the final dose for GAlbudAb and 30 min post the final dose for exendin-4. In a subgroup of animals, the final dose of exendin-4 was administered (1 μg/kg, SC, bid for 2 days) 6 h prior to myocardial ischemia when plasma exendin-4 was at its minimum concentration (C_min_). Myocardial infarct size, area at risk and cardiac function were determined 24 h after myocardial I/R injury.

**Results:**

GAlbudAb and exendin-4 significantly reduced myocardial infarct size by 28% and 23% respectively, compared to vehicle (both p < 0.01 vs. vehicle) after I/R injury. Moreover, both GAlbudAb and exendin-4 markedly improved post-ischemic cardiac contractile function. Body weight loss and reduced food intake consistent with the activation of GLP-1 receptors was observed in all treatment groups. However, exendin-4 failed to reduce infarct size when administered 6 h prior to myocardial ischemia, suggesting continuous activation of the GLP-1 receptors is needed for cardioprotection.

**Conclusions:**

Cardioprotection provided by GAlbudAb, a long acting GLP-1 mimetic, following myocardial I/R injury was comparable in magnitude, but more sustained in duration than that produced by short-acting exendin-4. Very low plasma concentrations of exendin-4 failed to protect the heart from myocardial I/R injury, suggesting that sustained GLP-1 receptor activation plays an important role in providing cardioprotection in the setting of acute myocardial I/R injury. Long-acting GLP-1 agonists such as GAlbudAb may warrant additional evaluation as novel therapeutic agents to reduce myocardial I/R injury during acute coronary syndrome.

## Introduction

Glucagon-like peptide-1 (GLP-1) is an incretin hormone secreted by intestinal L-cells in response to nutrient ingestion [1]. GLP-1 occurs endogenously in a number of biologically active isoforms including full length GLP-1 (7–36) amide, a glycine-extended isoform of GLP-1 (7–37), and the N terminus cleaved GLP-1 (9–36). GLP-1 regulates glucose homeostasis by stimulating insulin secretion, inhibiting glucagon secretion, delaying gastric emptying and promoting satiety [2]. Although the major physiological functions of GLP-1 are associated with glycemic control, increasing evidence indicates that GLP-1 may also play an important role in cardiovascular physiology [3]. It has been reported that GLP-1 receptors are expressed in both heart and coronary vasculature [4] and activation of GLP-1 receptors by agonists results in a range of cardiovascular effects including cardioprotection against myocardial ischemic injury both *ex vivo*[5-7] and *in vivo*[8-13].

Active GLP-1 (7–36) has a very short half-life of about 2 min following exogenous administration as it is rapidly cleaved and inactivated in circulation by the protease dipeptidyl peptidase-IV (DPP-IV) [14,15]. This short half-life limits its use as a therapeutic agent as demonstrated by the fact that clinical studies with exogenous GLP-1 are typically constrained to a continuous infusion dosing strategy [12,13]. Several GLP-1 receptor agonists, including exendin-4, have been identified exhibiting an extended plasma half-life. Exendin-4 is a 39 amino acid peptide originally derived from the saliva of the gila monster. It has been shown to have both insulinotropic and insulinomimetic effects mediated by activation of GLP-1 receptors [16,17]. Exendin-4 also limits myocardial infarct size both in large animal and human studies [18-20]. Although exedin-4 has a longer plasma half-life than native GLP-1 (60 min vs. 2 min) [21], it still requires twice daily injection to achieve anti-diabetic effects. Therefore, several other approaches have been developed to extend the serum half-life of GLP-1, such as, a fusion of DPP-IV resistant GLP-1 dimer with albumin (albiglutide) which has a long half-life (5 days in human) [22] and provides cardioprotection in a rat model of myocardial I/R injury [11]. Another approach fuses the GLP-1 “warhead” to small antibody binding domains (11–13 KDa) that have high affinity and specificity for human serum albumin (AlbudAbs) [23-27]. Here, we describe the creation of GAlbudAb, DOM7h-14 (a domain antibody, dAb) genetically fused with GLP-1 (7–36, A8G), with alanine at position 8 replaced by glycine to render the peptide DPP-IV resistant, resulting in a agent with prolonged half-life and pharmacological activity in a rat model of I/R injury. We also compare its cardioprotective action to exendin-4. We hypothesized that because of its extended half-life relative to exendin-4, GAlbudAb would produce an extended period of cardioprotection as compared with exendin-4.

## Materials and methods

### Generation of GAlbudAb

GAlbudAb was generated by DNA synthesis. Briefly, GLP-1 (7–36) peptide with an A8G mutation was put N terminal of a helical linker [28] that was N terminal of the DOM7h-14 AlbudAb [25]. This was cloned for expression into the PTT5 vector system. Expression was performed in transfected HEK293E cells. Following transfection, cells were cultured in Freestyle media (Invitrogen #12338-026) supplemented with 25 μg/ml G418 (PAA) and 0.001% (v/v) Pluronic (Invitrogen, #24040-032) at 37°C/5% CO_2_ at 125 rpm, and harvested after 120 h. Protein was purified from clarified supernatant by affinity capture using protein L coupled to NHS streamline resin (GE Healthcare) followed by cationic exchange chromatography (SP FF, GE Healthcare, #17-5157-01). The identity of the final material was assessed by intact mass analysis. Sample purity was assessed by reverse-phase HPLC.

### *In vitro* assays of GAlbudAb activity and its affinity to serum albumin

For analysis of GAlbudAb affinity for serum albumin, 500 resonance units of albumin were coupled to a CM5 Biacore chip and binding curves generated by flowing protein diluted in Biacore HBS-P + buffer at a range of concentrations of protein in the range 39 nM to 5 μM across the Biacore chip. Affinity (K_D_) was calculated by fitting on-rate and off-rate curves for traces generated in the range of the kDa for the dAb. For analysis of GLP-1 activity, GAlbudAb was tested for the ability to induce cAMP-driven luciferase production in Chinese Hamster Ovary cells stably transfected with both human GLP-1 receptor and a luciferase reporter gene containing 6 cAMP response elements within the promoter [29,30]. Data were normalized to non-treated control cells and analysed using Prism (GraphPad software) to fit non-linear regression curves and derive the EC_50_. The affinity of GAlbudAb for human, cynomolgous monkey and rat serum albumin was determined by BIAcore analysis. The fusion protein was passed over the surface of a CM5 chip coated with serum albumin and the equilibrium dissociation constant, K_D_, determined as a measure of affinity using the 1:1 interaction model in BiaEval software.

### Pharmacokinetic profile of GAlbudAb and exendin-4 following a single subcutaneous administration and simulation of multi-dose pharmacokinetics

Male Sprague–Dawley rats (8-week-old) purchased from Charles-River laboratory (Wilmington, DE) were used for the study. All animal studies were performed in compliance with the Guide for the Care and Use of Laboratory Animals as published by the US National Institutes of Health and were approved by the Institutional Animal Care and Use Committee of GlaxoSmithKline. Rats were administered GAlbudAb subcutaneously at a dose of 1 mg/kg, and blood samples were collected at 1, 2, 4, 8, 24, 48 and 72 h post dose. In exendin-4 PK group, rats were administered exendin-4 subcutaneously at a dose of 10 μg/kg, and blood samples were collected at 10 min, 30 min, 60 min, 2, 4 and 8 h post dose. Based on the plasma concentrations of GAlbudAb at the dose of 1 mg/kg, a pharmacokinetic model was developed and parameters were estimated using WinNonlin version 5.1 (Pharsight Corporation). Computer simulations were also performed using WinNonlin version 5.1 to project the required dose for the *in vivo* cardioprotection study. GAlbudAb doses for the rat I/R injury experiment were selected with the goal that trough concentrations should be above 1000 ng/ml. A target of > 1000 ng/ml was based on previous *in vitro* efficacy data showing GLP-1 receptor activation. Similarly, PK modeling analysis for exendin-4 was performed to project maximum concentration (C_max_) and minimum concentration (C_min_) following multiple doses of 1 μg/kg subcutaneous administration.

### Myocardial ischemia/reperfusion injury, LV hemodynamic measurement and postmortem analysis in Sprague–Dawley rats

Sprague–Dawley rats were treated with vehicle or with GAlbudAb at doses of 0.6 mg/kg, 2 mg/kg and 6 mg/kg once daily by subcutaneous injection for two days or with exendin-4 (Phoenix Pharmaceuticals, INC) at 0.1 μg/kg, 1 μg/kg and 10 μg/kg, twice daily by subcutaneous injection for two days. The timing of the final dose was determined by the PK modeling to obtain C_max_ during the early reperfusion period. The final dose was administered 2 h prior to myocardial ischemia for GAlbudAb and 30 min prior to myocardial ischemia for exendin-4. Then, rats were subjected to 30 min myocardial ischemia followed by 24 h reperfusion as previously described [11,31,32]. In order to determine whether the GLP-1 target needed to be engaged continuously to provide cardioprotection, myocardial I/R injury was evaluated 6 h after exendin-4 treatment (1 μg/kg) using the same dosing regimen as above at the time when plasma concentrations of exendin-4 was at C_min_. LV hemodynamic and myocardial infarct size was determined as previously described [11,24,25]. Briefly, at the end of study, rats were anesthetized with 2% isoflurane in oxygen and a 2 F Millar Mikro-tip catheter transducer was inserted into the left ventricle through the right carotid artery to measure left ventricular pressure and both systolic and diastolic left ventricular function (dP/dt_max_ and dP/dt_min_, respectively). After hemodynamic measurements, the heart was excised and perfused with saline to wash out residual blood through an aortic cannula. To delineate infarcted tissues from viable myocardium, the heart was then perfused with a 1% solution of 2,3,5-triphenyltetrazolium chloride (TTC) in phosphate buffer (pH 7.4, 37°C). The viable myocardium was stained red, and the infarcted myocardium was stained white. To delineate the area at risk (ischemic area), the coronary artery was then tied at the site of the previous occlusion and the aortic root was perfused with a 1% Evans blue dye (Sigma) in normal saline. As a result of this procedure, the portion of the LV supplied by the previously occluded coronary artery (area at risk) was identified by the absence of blue dye, whereas the rest of the LV was stained dark blue. The heart was frozen, after which all atrial and right ventricular tissues were excised. The LV was cut into 4–5 transverse slices, which were photographed using a digital camera. The borders of the infarct, area at risk and area of non at risk of heart images were traced and measured using Image-Pro Plus. Area at risk and infarct size was calculated using the following mathematical formulas: Area at risk = sum of area at risk/(sum of area at risk + sum of area not at risk) × 100%; infarct size = (sum of infarct area/sum of area at risk) × 100%.

### Biochemical analysis, clinical chemistry, exendin-4 and GAlbudAb bioanalysis

Plasma insulin was measured using an MDS rat insulin Kit (K152BZC-2). Plasma glucose and lactate were measured in non-fasted animals using an Olympus AU 640 analyzer (Olympus America Inc., Melville, NY). Plasma levels of exendin-4 were measured using an exendin-4 ELISA kit (Bachem, San Carlos, CA). Plasma GAlbudAb concentrations were quantified using an analytical method based on sample dilution followed by LC-MS/MS analysis. Briefly, working solutions were prepared from the GAlbudAb stock solution at 1, 10 and 100 μg/mL in rat plasma. From these working solutions, a standard curve was prepared with nominal concentration of 100, 250, 500, 1000, 2500, 5000 and 10000 ng/mL in rat plasma. Then, samples, standards, QC samples and blanks were aliquoted (50 μL) into separate 1.4 mL polypropylene tubes. This was followed by digestion using 75 μL of Lys-C solution (1 IU of Lys-C/mL of 100 mM sodium bicarbonate buffer pH 8.5) and incubated overnight at 37°C with constant shaking. To stop the digestion samples were acidified with formic acid and subjected to solid phase extraction using Strata XC strong cation exchange resin (Phenomenex) according to the manufacturer’s peptide extraction procedure. The resultant peptides were resuspended in 90/10/0.1 water/acetonitrile/formic acid for LC-MS/MS analysis. Peptides were separated on an Acquity BEH-C18 1.7 μm 2.1 × 50 mm column with a column temperature held at 65°C and a flow rate of 0.7 mL/min. HPLC-MS/MS analyses were performed on a triple quadrupole mass spectrometer API5000 (Applied Biosystems/MDS Sciex) with a TurboIonSpray electrospray ionization source equipped with a Waters Acquity UPLC system. MS data was acquired and processed using the proprietary software application Analyst version 1.4.2 (Applied Biosystems/MDS Sciex). Calibration plots for the LC-MS/MS assay of analyte/IS peak area ratio were plotted versus concentration and a weighted 1/*x*^2^ linear regression was applied to the data.

### Statistical analysis

Data are presented as mean ± SEM. Differences between groups were compared by paired and unpaired Student’s t tests or by a one-way ANOVA followed by a Bonferroni test for multiple comparisons. A p value of < 0.05 was considered statistically significant.

## Results

### Characterization, expression and *in vitro* activity of GAlbudAb

**Figure 1:**
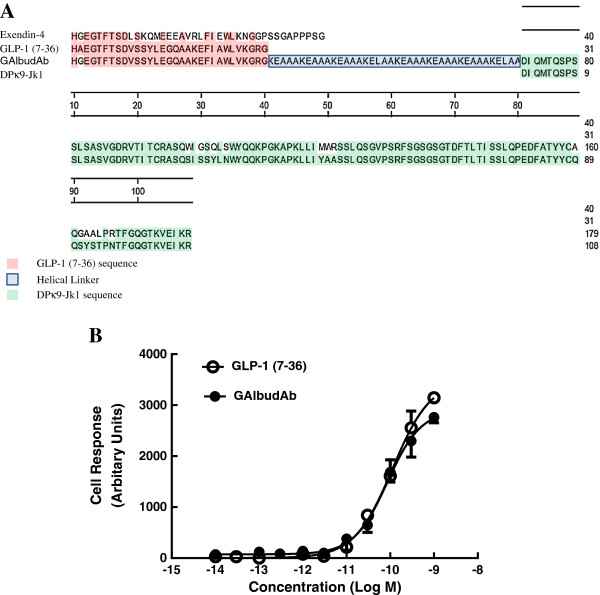
**Amino acid sequence of GAlbudAb and activity of GAlbudAb.** The sequence was aligned with that of the amino acid sequence for the human DPK9 - Jk1 germline gene, the GLP-1 (7–36) peptide and exendin-4 using the Clustal V algorithm. Residue numbering was determined by the method of Kabat **(A)**. Activity of native GLP-1 (7–36) and GAlbudAb in CHO reporter assay **(B)**.

GAlbudAb fusion protein was cloned into a pTT5-based expression vector [26], transfected into HEK293E cells and purified directly from tissue culture supernatant following 5 days culture. Alanine at position 8 of GLP-1 (7–36) was replaced with glycine to render it DPP-IV resistant. The amino acid sequences of the fusion protein and its alignment to GLP-1 (7–36) peptide, exendin-4 and the germ-line DPκ9-JK1 sequence are shown in Figure 1A. Purified protein produced a single band of expected mass on SDS-PAGE as well as the expected mass by mass spectrometry (data not shown).

To determine whether the purified fusion protein was active, GLP-1 activity was assayed using a GLP-1 receptor CHO reporter assay (Figure 1B) in which CHO cells are stably transfected with a GLP-1 receptor gene and a luciferse reporter gene under the control of a 6 cAMP response element promoter. Treatment of this cell line with GLP-1 receptor agonists results in induction of reporter gene expression in a manner dependent upon the level of GLP-1 receptor activation. The GLP-1(7–36) peptide and the fusion protein induced reporter gene expression in this cell line with equivalent potency (EC_50_ = 100 pM). Affinity of the fusion protein for human, cynomolgus and rat serum albumin was approximately 143, 395 and 275 nM, respectively.

### Pharmacokinetics of GAlbudAb following a single subcutaneous injection in Sprague–Dawley rat

**Figure 2:**
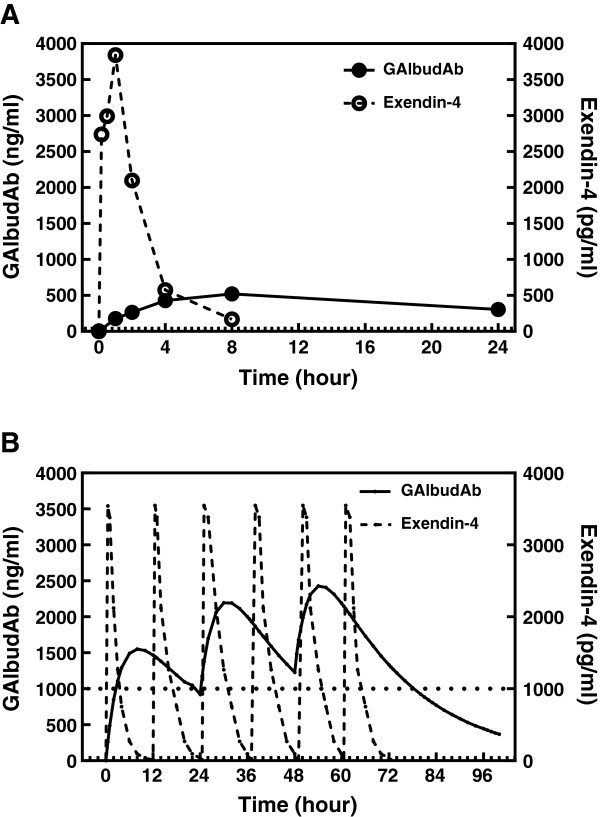
**Rat GAlbudAb and exendin-4 pharmacokinetic analysis and computer simulation for predicting plasma drug concentrations. A**, Plasma GAlbudAb concentrations following 1 mg/kg subcutaneous administration and blood samples were collected at 1, 2, 4, 8 and 24 h and plasma exendin-4 concentrations following 10 μg/kg subcutaneous administration and blood samples were collected at 10 min, 30 min, 60 min, 2, 4 and 8 h (n = 3/group). **B**, Computer simulated GAlbudAb concentration curves at a projected dose of 2 mg/kg, which achieved a sustained exposure above 1000 ng/ml after 48 h and computer simulated exendin-4 concentration curves following 1 μg/kg twice daily SC dosing of exendin-4 for 3 days, which showed exendin-4 was at its C_min_ 6 h post dose.

Rat plasma concentrations of GAlbudAb and exendin-4 (Figure 2A) following a single subcutaneous injection (1 mg/kg and 10 μg/kg, respectively) were determined using LC-MS/MS analysis and ELISA assay. The time to peak concentration for GAlbudAb was 8 h and the maximum plasma concentration for GAlbudAb was 523 ± 98 ng/ml as determined by LC-MS/MS. GAlbudAb was detected in circulation for at least 24 h. The time to peak concentration for exendin-4 was 1 h and the maximum plasma concentration for exendin-4 was 3843 ± 649 pg/ml as determined by ELISA.

Based on this initial pharmacokinetic profiling, a computer simulated analysis was performed to project plasma concentrations of GAlbudAb and exendin-4 following multiple days dosing and to determine the dose required for the *in vivo* cardioprotection study. As shown in Figure 2B, simulated plasma concentrations of GAlbudAb following a subcutaneous injection at a dose of 2 mg/kg/day for 3 days were above 1000 ng/ml, the target plasma concentration for the *in vivo* study. In contrast to GAlbudAb, simulated plasma concentrations revealed that exentin-4 was at its C_min_ 6 h post the final dose (Figure 2B). Final dose selection for GAlbudAb bracketed this target level and contained a one-log concentration range. Dose selection for exendin-4 was based on previous studies [18,33].

### Myocardial infarct size and area at risk following ischemia/reperfusion injury

**Figure 3:**
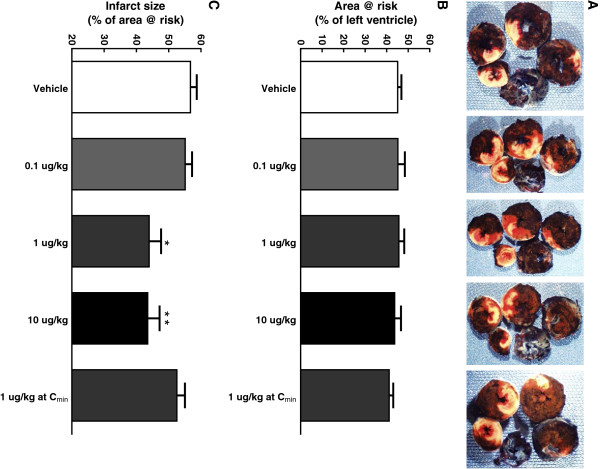
**Effect of exendin-4 on infarct size and area at risk following acute myocardial ischemia/reperfusion injury.** Sprague–Dawley rats were subjected to 30 min LAD coronary artery occlusion followed by 24 h reperfusion. Hearts were harvested and analyzed for area at risk and infarct size. Representative photographs of heart sections from vehicle, 0.1, 1, 10 μg/kg and 1 μg/kg exendin-4 at C_min_ (from left to right) stained by 2,3,5-triphenyltetrazolium chloride (TTC) and Evans blue dye illustrate myocardial infarct (white), area at risk (white and red) and area not at risk (dark blue) **(A)**. Myocardial infarct size was assessed as percentage of area at risk in the left ventricle **(B)**. The area at risk was assessed as percentage of the left ventricle area **(C)**. Values are presented as mean ± SEM. *p < 0.05 and ^**^p < 0.01 vs. vehicle (n = 8-10/group).

A total of 72 rats were subjected to 30 min ischemia and 24 h reperfusion and 70 rats survived the procedure. One rat from the vehicle group died at 2 h post reperfusion and one rat from the GAlbudAb low dose group (0.6 mg/kg/day) died overnight after reperfusion. Representative transverse sections of the heart stained by TTC and Evans blue from each group are shown in Figure 3A. Myocardial infarct size averaged 56.8 ± 2.0% of area at risk in the vehicle group after myocardial I/R injury while treatment with exendin-4 at the doses of 0.1, 1 or 10 μg/kg reduced infarct size to 55.1 ± 2.1%, 44.0 ± 3.7% and 43.5 ± 3.7% of area at risk (reduced by 2.9%, 22.5% and 23.3% vs. vehicle, respectively) (Figure 3C). Infarct size was 52.5 ± 2.5% of area at risk (i.e. comparable to infarct size following vehicle treatment) in the exendin-4 group treated 6 h prior to myocardial ischemia at the C_min_ (Figure 3C). Area at risk was comparable across all groups (Figure 3B).

**Figure 4:**
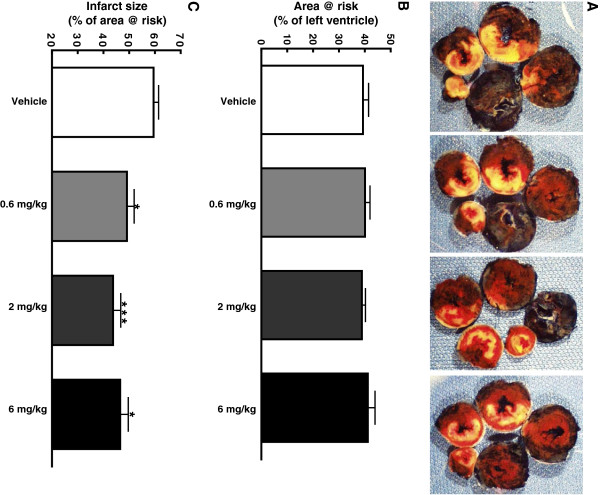
**Effect of AlbudAb on infarct size and area at risk following acute myocardial ischemia/reperfusion injury.** Sprague–Dawley rats were subjected to 30 min LAD coronary artery occlusion followed by 24 h reperfusion. Hearts were harvested and analyzed for area at risk and infarct size. Representative photographs of heart sections from vehicle, and 0.6, 2, or 6 mg/kg GAlbudAb treated group (from left to right) stained by 2,3,5-triphenyltetrazolium chloride (TTC) and Evans blue dye illustrate myocardial infarct (white), area at risk (white and red) and area not at risk (dark blue) **(A)**. Myocardial infarct size was assessed as percentage of area at risk in the left ventricle **(B)**. The area at risk was assessed as percentage of the left ventricle area **(C)**. Values are presented as mean ± SEM. *p < 0.05 and ***p < 0.001 vs. vehicle (n = 4-9/group).

Treatment with GAlbudAb significantly reduced infarct size. Infarct size was 49.1 ± 2.8%, 43.7 ± 3.0%, and 46.5 ± 3.1% of area at risk at doses of 0.6, 2 or 6 mg/kg (reduced by 19%, 28% and 24% vs. vehicle, respectively, all p < 0.05) (Figure 4A and C). Area at risk was comparable between vehicle- and GAlbudAb-treated groups (Figure 4B).

### Cardiac function following ischemia/reperfusion injury

There was no difference in heart rate or LV systolic pressure between vehicle- and GAlbudAb-treated animals (Table 1). However, the increased LV end diastolic pressure induced by myocardial I/R injury was markedly attenuated by GAlbudAb (8.6 ± 1.5 mmHg vs. 12.8 ± 1.0 mmHg in vehicle, p < 0.05) (Table 1). Additionally, the impaired contractile and relaxation function post-myocardial I/R injury were significantly improved with GAlbudAb treatment by 14.8% and 18.7% respectively, compared to vehicle (7612 ± 219 mmHg/s vs. 6628 ± 273 mmHg/s for dP/dt_max_ and 6746 ± 341 mmHg/s vs. 5682 ± 311 mmHg/s for dP/dt_min_) (Table 1). Exendin-4 also enhanced post-ischemic cardiac function (data not shown).

**Table 1:** Effect of GAlbudAb on post-ischemic left ventricle hemodynamic

**Parameter**	**Vehicle**	**GAlbudAb (2 mg/kg)**
N	8	9
LVSP (mmHg)	107 ± 3.0	112 ± 2.6
LVEDP (mmHg)	12.8 ± 1.0	8.6 ± 1.5*
HR (bpm)	425 ± 5	419 ± 10
dP/dt_max_ (mmHg/s)	6628 ± 273	7612 ± 219*
dP/dt_min_ (mmHg/s)	5682 ± 311	6746 ± 341*

N, number of animals; LVSP, left ventricle systolic pressure; LVEDP, left ventricle end diastolic pressure; HR, heart rate; dP/dt_max_, maximum rates of change in the left ventricle pressure; dP/dt_min_, minimum rates of change in left ventricle pressure. Data are presented as mean ± SEM. *p < 0.05 vs. vehicle.

### Body weight, food consumption, and plasma levels of insulin, glucose, lactate and GAlbudAb concentrations pre-ischemia and 24 hrs post-reperfusion

**Figure 5:**
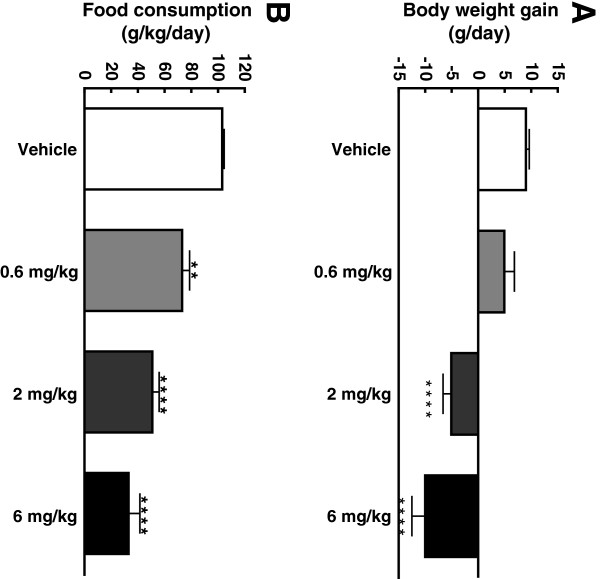
**Body weight gain (A) and food consumption (B) after 2 days GAlbudAb treatment.** Values are presented as mean ± SEM. **p < 0.01 and ****p < 0.0001 vs. vehicle.

**Figure 6:**
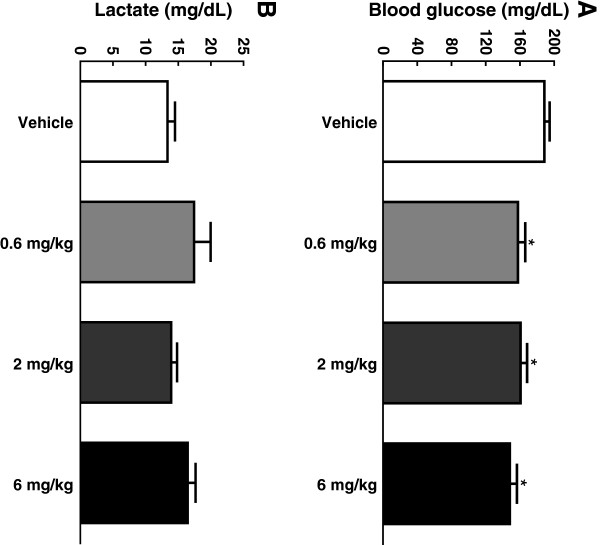
**Blood glucose (A) and blood lactate (B) after 2 days GAlbudAb treatment.** Values are presented as mean ± SEM. *p < 0.05 vs. vehicle.

GAlbudAb significantly reduced body weight and food consumption in a dose-dependent manner following two days of dosing, which is consistent with the known pharmacology of GLP-1 mimetics (Figure 5A and B). GAlbudAb also decreased pre-ischemia plasma glucose compared to vehicle at all doses (Figure 6A). There was no difference in plasma lactate between GAlbudAb and vehicle-treated rats (Figure 6B). Plasma concentrations of GAlbudAb prior to a 30 min myocardial ischemia were 2476 ± 561 ng/ml and 2606 ± 175 ng/ml at the doses of 2 mg/kg and 6 mg/kg, respectively, which were above the projected drug exposure to activate GLP-1 receptors when myocardial I/R injury occurred. The terminal plasma concentrations of GAlbudAb 24 h post reperfusion were 465 ± 69 ng/ml and 1667 ± 611 ng/ml for the doses of 2 mg/kg and 6 mg/kg, respectively.

## Discussion

In this study, we demonstrate that fusion of a domain antibody with high affinity and specificity for human serum albumin (AlbudAb) to a DPP-IV resistant GLP-1 (7–36, A8G) peptide produced a potent GLP-1 agonist with an extended plasma half-life and cardioprotective activity in an acute model of myocardial I/R injury. The cardioprotective effects of GAlbudAb were further confirmed by improvement in post-ischemic cardiac function. The cardioprotective effects of GAlbudAb were equiefficacious when compared to exendin-4 and were consistent with the known effects of GLP-1 receptor activation, i.e. dose-dependent reduction in body weight, food intake and plasma glucose. However, GAlbudAb provided an infarct-limiting effect following myocardial I/R injury that was of similar magnitude, but longer duration than that produced by exendin-4, which failed to protect the heart against I/R injury at 6 h post dosing when plasma exendin-4 concentrations were projected to be very low and considerably below concentrations previously shown to be necessary for GLP-1 receptor activation. In addition, the known pharmacologic effects of activation of GLP-1 receptor by GAlbudAb were verified in this study; GAlbudAb dose-dependently reduced animal body weight gain, which was associated with a reduction in rat food intake. A glucose lowering effect of GAlbudAb was also observed in the present study. The observed pharmacological effects of GAbuldAb are consistent with the stability of this construct *in vitro* and *in vivo* (Figure 1B and Figure 2A). These findings suggest that in addition to providing peripheral glycemic control, GAlbudAb may have direct therapeutic potential for limiting myocardial infarct size and improving cardiac function in the setting of acute myocardial ischemic injury.

GLP-1 and its various mimetics have been shown to be cardioprotective in the setting of myocardial I/R injury [10,11]. Timmers et al. reported exenatide, a synthetic version of exendin-4, reduced myocardial infarct size by 40% in a porcine model of myocardial I/R injury [18]. More recently, Lonborg et al. demonstrated that exenatide reduces final infarct size in patients with ST-segment-elevated myocardial infarction and a short-duration of ischemia [20]. In line with these previous findings, we confirmed the infarct-size limiting effects of exendin-4 in our rat model of myocardial I/R injury. We also explored the cardioprotective effects of exendin-4 following twice daily subcutaneous administration when plasma drug concentrations were very low. Exendin-4 failed to demonstrate an infarct size limiting effect when plasma exendin-4 at its C_min_, indicating that a certain level of plasma exposure of exendin-4 is critical to protect the heart from myocardial I/R injury and to activate the cardiac GLP-1 receptor. In contrast to exendin-4, GAlbudAb has a longer serum half-life. Plasma concentrations of GAlbudAb during 30 min ischemia and early reperfusion period (2476 ± 561 ng/ml) were above the required threshold exposure to activate GLP-1 receptors (1000 ng/ml), and a substantial concentration of GAlbudAb in circulation was detected even 26 h post final dose (1667 ± 611 ng/ml). Therefore, it can be postulated that GAlbudAb would result in increased duration of cardioprotection versus exendin-4 in our rat model of myocardial I/R injury. However, maximal cardioprotection between GAlbudAb and exendin-4 was similar (28% vs. 23% of myocardial infarct reduction, respectively). These findings are further supported by clinical data in diabetic patients in which sustained pharmacology has demonstrated greater efficacy in term of glycemic control [34,35].

Several mechanisms by which GLP-1 and its mimetics render cardioprotection have been previously identified [18,36-41]. Timmers et al. have shown that exenatide reduces myocardial infarct size, decreases oxidative stress and inhibits caspase-3 expression and DNA fragmentation in a porcine model of myocardial I/R injury [18]. Ye et al. have demonstrated that increases in cAMP, PKA and activation of Akt by exendin-4 contribute to the cardioprotection by exendin-4, which results in enhanced post-ischemia cardiac function [38]. More recently, we demonstrated that increases in glucose metabolism and a shift toward a more energetically favorable substrate metabolism by increasing both glucose and lactate oxidation also play an important role in GLP-1 analog mediated cardioprotection in the rat [11]. Furthermore, Ku et al. reported DPP4 deficient rats exhibited improved cardiac function following myocardial I/R injury via activation of GLP-1 receptor and its downstream signaling [39]. Myocardial I/R injury initiates an inflammatory cascade and a recent study revealed that cytoprotection against hypoxia produced by exendin-4 in human islets was associated with inhibition of inflammatory genes through activation of cAMP response element binding protein [41]. However, it appears cardioprotection produced by GAlbudAb is in large part due to direct cardiomyocyte protection because GAlbudAb not only reduced myocardial infarct size but also significantly enhanced post-ischemia LV function. Xiao et al. demonstrated that GLP-1 increased cardiomyocyte contractility through enhancing cardiac L-type Ca^2+^ current via activation of the cAMP-deperndent protein kinase A pathway [40]. It is possible that these mechanisms are also involved in the cardioprotection mediated by GAlbudAb, but the precise mechanism by which cardioprotection is rendered by GAlbudAb warrants future research. Previous studies also suggest that cardioprotection might be elicited by GLP-1 (9–36) via GLP-1 receptor independent pathways [10]. However, GLP-1 (9–36) AlbudAb (data not shown), didn’t significantly reduce myocardial infarct size in this study and it also had no effect on body weight and food intake following two days of treatment, suggesting activation of the GLP-1 receptor dependent pathway by GAlbudAb plays an important role in cardioprotection. In addition, the GLP-1 (7–36, A8G) engineering rendered the peptide DPP-IV resistant thereby limiting the amount of GLP-1 (9–36) generated *in vivo*. These results were consistent with GLP-1 receptor activation. Although GLP-1 receptors have been identified in cardiomyocytes and endothelial cells, an indirect benefit to the heart via systemic glucose/insulin regulation cannot be ruled out.

In this study, we demonstrate that GAlbudAb produced both glucose-lowering and cardioprotective effects, suggesting that GAlbudAb may have potential both to manage hyperglycemia and to limit myocardial ischemia injury in patients with diabetes mellitus who are at high risk for coronary artery disease and acute coronary syndrome. Moreover, the results demonstrate that extended plasma exposure and resulting extended period of GLP-1 receptor activation produced by GAlbudAb prolonged the period of ischemic protection. Additional studies would be required to determine whether this observation would be translated into a greater period of protection from ischemic injury in patients with diabetes mellitus and coronary artery disease.

## Competing interests

All authors are employees of GlaxoSmithKline.

## Authors’ contributions

WB, LJH, AB, LJ, JJL, RNW, ED and BMJ conceived and designed the experiments; WB, LJH, RP, GJ, MS, KA performed the experiments; WB, LJH, RP, GJ, MS and BMJ analyzed the data; WB, BJM, RNW and JJL wrote the paper. All the authors read and approved the manuscript.
